# Social Bots’ Sentiment Engagement in Health Emergencies: A Topic-Based Analysis of the COVID-19 Pandemic Discussions on Twitter

**DOI:** 10.3390/ijerph17228701

**Published:** 2020-11-23

**Authors:** Wen Shi, Diyi Liu, Jing Yang, Jing Zhang, Sanmei Wen, Jing Su

**Affiliations:** 1 Department of Earth System Science, Tsinghua University, Beijing 100084, China; shi-w18@mails.tsinghua.edu.cn; 2 School of Journalism and Communication, Renmin University of China, Beijing 100084, China; diyi.liu@ruc.edu.cn; 3 School of Journalism and Communication, Tsinghua University, Beijing 100084, China; yangjing17@mails.tsinghua.edu.cn (J.Y.); zjing20@mails.tsinghua.edu.cn (J.Z.); 4 Center for International Communication Studies, Tsinghua University, Beijing 100084, China; 0163100462@shisu.edu.cn; 5 School of Humanities, Tsinghua University, Beijing 100084, China

**Keywords:** social bots, social media, sentiment analysis, COVID-19 pandemic, health emergency

## Abstract

During the COVID-19 pandemic, when individuals were confronted with social distancing, social media served as a significant platform for expressing feelings and seeking emotional support. However, a group of automated actors known as social bots have been found to coexist with human users in discussions regarding the coronavirus crisis, which may pose threats to public health. To figure out how these actors distorted public opinion and sentiment expressions in the outbreak, this study selected three critical timepoints in the development of the pandemic and conducted a topic-based sentiment analysis for bot-generated and human-generated tweets. The findings show that suspected social bots contributed to as much as 9.27% of COVID-19 discussions on Twitter. Social bots and humans shared a similar trend on sentiment polarity—positive or negative—for almost all topics. For the most negative topics, social bots were even more negative than humans. Their sentiment expressions were weaker than those of humans for most topics, except for COVID-19 in the US and the healthcare system. In most cases, social bots were more likely to actively amplify humans’ emotions, rather than to trigger humans’ amplification. In discussions of COVID-19 in the US, social bots managed to trigger bot-to-human anger transmission. Although these automated accounts expressed more sadness towards health risks, they failed to pass sadness to humans.

## 1. Introduction

Social media is an emerging platform for the public to find information, share opinions, and seek coping strategies when faced with health emergencies [1,2,3]. In a disease outbreak, sentiments and emotions from social media data are regarded as useful mental health indicators [4]. Strong emotions, such as anxiety, fear, and stress about the disease, might be triggered by updates on infection and mortality and be shared on social media [5]. In turn, health information, telemedicine, and online psychological counseling on social media can help relieve individuals’ mental pressure. Interactions have also been found to promote emotion transmission on social media [6,7], potentially affecting individuals’ cognition and behaviors [8].

In the coronavirus disease 2019 (COVID-19) outbreak, a global health crisis caused by the infectious coronavirus, the public’s reliance on online information climbed [9,10] in order for people to remain informed and connected as a result of a series of infection control measures such as travel limitations, community quarantine, and social distancing. Researchers found that people expressed their fears of infection and shock regarding the contagiousness of the disease [11], along with their feelings about infection control strategies, on social media [12,13,14]. They also showed emotional reactions towards some health-unrelated themes, such as the economy and the global environmental impact of the COVID-19 pandemic [15]. Meanwhile, racist conspiracies and hateful speech concerning the pandemic [16], which have been proved to provoke negative emotions [17], also proliferated on social media. More social media exposure may have resulted in a higher probability of anxiety and depression [18].

The abovementioned studies on online sentiments during health emergencies assumed that all the content of the digital ecosystem was produced by humans. However, established evidence has proven that social bots—accounts controlled by computer algorithms [19]—can prosper on social media and be involved in online sentiment manipulation [20]. These automated accounts can increase users’ exposure to negative and inflammatory messages, triggering mass hysteria and social conflicts in cyberspace [21]. They may also intentionally pick sides and disseminate a more complex set of emotions to attract more attention [22].

In health-related topics, social bots have been accused of promoting polarized opinions and misleading information [23,24,25]. Scientists have identified social bots in online communication around the COVID-19 pandemic and demonstrated their advocacy of conspiracies [23]. Nevertheless, social bots’ sentiment engagement in the pandemic has not been empirically studied. As Twitter may potentially be used by health experts and policy makers to understand the opinions of the general public [14,26], investigating social bots’ interference in sentiments may lay the groundwork for accurate policy-making and strategical communication in health emergencies. This study aimed to characterize social bots’ sentiment engagements during the COVID-19 pandemic. We selected three important timepoints in the progress of the COVID-19 pandemic and conducted a topic-based sentiment analysis of bot-generated and human-generated tweets to figure out social bots’ sentiment expressions and transmission.

## 2. Literature

### 2.1. Emotions and Public Health Emergencies on Social Media

In public health emergencies, such as the COVID-19 pandemic, the increasing number of infected people and rising mortality elicited strong negative emotions such as anxiety, anger, and sadness in the public [5,27]. Prevention measures, such as quarantine and travel bans, limited individuals’ freedom, thus further raising people’s negative emotions [28,29]. Health-unrelated factors like the economic and political impact, media coverage, and government responses also served as drivers of expressions of emotion [30,31]. However, it is worth noting that during public health emergencies, if there is no better or more convenient offline way to vent emotions, it is expected that people will turn to social media [1,2,3,32,33]. In addition to offering medical information and experiences that people may find useful [34,35,36,37], social media also accommodates the need for emotional release. Recent studies on emotions expressed on social media in the COVID-19 pandemic in various countries found negative emotions like depression, anxiety, fear, and anger increased and became more prevalent [4,11,38,39]. Further, since the impacts of geographical boundaries and offline interpersonal circles are attenuated on social media, emotions can be shared more easily and affect others’ emotions more widely, gradually forming a homogeneous emotional state, the process of which is termed “emotion contagion” [29,40].

### 2.2. Emotional Contagion on Social Media

Emotion contagion describes a phenomenon in which certain individuals’ emotions propagate to others and trigger similar ones; this may take place in both the online and offline worlds [41,42,43]. In recent years, especially with the development of social media, a growing number of studies on emotion contagion have shifted their attention to this new arena [6,7,31]. It has been found that although people on social media cannot encounter non-verbal cues conveying emotions, which are common in face-to-face communication, emotion contagion could still occur via online emotional content [6,7,31,44]. Plus, on social media, the scope of people’s interactions is larger than their social circles in reality [29]. It should also be noted that now, on social media, not only does emotion contagion take place in human-human interactions, but also in human-robot interactions [45].

Complex factors affect emotion contagion on social media. Firstly, the valence of the content; although the academia has not reached consensus on whether positive or negative contents can be shared more on social media, it is clear that emotional content travels faster than non-emotional content [29]. Furthermore, specific to different types of emotions, some emotions in social media messages are more likely to arouse public sharing behaviors than others, such as anger [46]. Contagion of negative emotions, like anger, sadness, and anxiety, on social media may have been accelerated during the COVID-19 pandemic [29]. For example, the “Purell panic”, stating that out of fear of being infected by the virus, people would rush to buy Purell irrationally, in the coronavirus crisis is a convincing example [47]. Furthermore, people’s emotions are susceptible to manipulation by other users or even bots [23,43,48] and their influence can be exacerbated in certain topics related to disease outbreaks [31,49].

### 2.3. Social Bots and Public Sentiments Manipulation

While mostly acting in a coordinated fashion, social bots—accounts that are in part or entirely controlled by computer algorithms rather than human users [19]—have been found to drive public emotional and sentiments dynamics in social media conversations [19,22,50,51]. Over the past few years, social bots have become increasingly prevalent on social media platforms, such that they now comprise approximately 9–15% of active Twitter accounts [20]. In the Twittersphere, social bots can drive high volumes of traffic, amplify polarizing and conspiracy content [52,53], and contribute to the circulation of misinformation [54].

By analyzing 7.7 million tweets in the 2014 Indian election, scholars found that bots tend to be less polarized when expressing sentiments—either positive or negative—than human users and are less likely to change their stance [55]. However, this does not necessarily indicate that social bots are more neutral in nature. Instead, they are often involved in public sentiment manipulation. Since emotionally charged tweets tend to be retweeted more often and more quickly than neutral ones [56], during polarizing events, social bots intentionally pick sides and disseminate a more complex set of emotions to attract more attention, while human users commonly express basic emotions, including sadness and fear [22]. These automated accounts can also increase users’ exposure to negative and inflammatory messages, which may trigger mass hysteria and social conflicts in cyberspace [21]. In spam campaigns, social bots are employed in an orchestrated fashion to post negative tweets, so as to smear competing products [57]. They can elicit varied emotional reactions, ranging from worship to hatred, during interactions with human users [58]. Even on harmless occasions such as Thanksgiving, these accounts are apt to trigger polarizing emotions in Twitter discourse [22].

### 2.4. Social Bots and Online Health Communication

Social bots may also be used for manipulating public attitudes, sentiments, and behaviors by engaging in online health-related discussions. On the one hand, through effective and friendly interactions with human users, social bots can assist people with achieving their physical and mental health goals [59], such as through controlling tobacco use [60] and providing pediatric groups with mental health care, education, and consultations [61]. In the fight against the COVID-19 pandemic, researchers have noted that chatbots could be used to spread up-to-date health information, ease people’s health anxiety around seeking medical care, and alleviate psychological pain [62]. On the other hand, in online social networks, some malicious bots pose multifaceted threats to public health. For example, in vaccine controversies, social bots can be exploited to alter people’s willingness in vaccination uptake and strengthen anti-vaccination beliefs through retweeting content posted by users in this opinion group [63]. Furthermore, sophisticated automated accounts, like Russian trolls, strategically pay equal attention to pro- and anti-vaccination arguments; this is a well-acknowledged tactic to disrupt civic conversations, which has effectively tampered with public confidence in vaccine efficacy [24]. In addition, by suggesting medical uses for cannabis, social bots can also be employed to skew the public’s attitudes towards the efficiency of cannabis in dealing with mental and physical problems [25].

During earlier public health emergencies, as shown in the Zika epidemic debate, social bots were detected as both influential and dominant voices on social media [64]. Some recent works have also investigated social bots’ engagement in online conversation pertaining to COVID-19. Researchers from Carnegie Mellon University (CMU) found that many social bots spread and amplified false medical advice and conspiracy theories and in particular, promoted “reopening America” messages [65]. These automated agents have become even more active in the era of COVID-19, doubling the activity with respect to researchers’ estimates based on previous crises and elections [65,66].

It has been well acknowledged that in the outbreak, non-human accounts contributed to social media infodemics [23,66,67], yet bots’ involvement in public sentiments during the pandemic remains unclear. In particular, social bots’ probable amplification of negative emotions, as well as their success and failure in triggering humans’ amplification under distinct sub-topics of health emergencies, have not been examined. This paper seeks to paint a mixed picture of the Twitter discourse during COVID-19 when a diverse range of users shared their concerns, expressed emotions, and transmitted their sentiments and feelings in different ways, with a particular focus on characterizing the role of social bots in the online information ecosystem. To be specific, this study aims to address the following questions:

RQ1 When discussing different topics concerning the COVID-19 pandemic, what are the differences between social bots and human users in terms of their (a) sentiment polarity and (b) sentiment strength?
RQ2 For those topics showing negative sentiments, what are the differences between social bots and human users in terms of their expressions of specific negative emotions, including (a) anger, (b) anxiety, and (c) sadness?
RQ3 Will (and if so, how) the negative emotions in tweets be transmitted among different actors?

## 3. Materials and Methods

### 3.1. Data

In this study, we followed previous research [23,68,69] and chose Twitter to explore the sentiment features of social bots during the COVID-19 pandemic. According to the COVID-19 pandemic timeline revealed by the WHO [70], we selected three important time points relating to the WHO’s reactions as data collection timestamps. (1) On 22 January, the WHO mission to Wuhan issued a statement saying that evidence suggested human-to-human transmission in Wuhan, but more investigation was needed to understand the full extent of transmission, which marks the WHO’s attention to and assessment of the potential spread of COVID-19. (2) The WHO declared the novel coronavirus a public health emergency of international concern (PHEIC) on 31 January, indicating the worldwide concern related to COVID-19. (3) The WHO made the assessment that the COVID-19 outbreak could be characterized as a pandemic on 11 March, which meant the world was in the grip of COVID-19.

Although on 11 February, the WHO announced that the disease caused by the novel coronavirus was called COVID-19 (short for Corona Virus Disease 2019), our data collection time points span from January to March. Considering the time points, we used #coronavirus (case insensitive) as a data collection keyword to ensure consistency.

This study used a self-designed Python script to collect tweets and authors. We followed Twitter’s robot policy and only collected publicly available tweet texts in this study. Twitter users’ privacy was totally respected. The following kinds of tweets were included: (1) all original tweets containing the keyword and (2) Quote tweets containing the keyword in the comment part. In other words, all novel contents containing “#coronavirus” were collected. Whereas, quote tweets of which their comment did not contain the keyword and retweets were not included. With this search criteria, we made sure that all the texts with the keyword “#coronavirus” were posted by the documented users. Emojis, symbols, and hyperlinks in the text were filtered out. Twitter users’ privacy was totally respected. Personal information was not collected, analyzed, or displayed in our research.

### 3.2. Social Bot Detection

Given that social bots have become increasingly sophisticated, the lines between human-like and bot-like behaviors have become further blurred [19]. Researchers have developed multiple approaches to discriminate between social bots and humans [71,72], among which *Botometer*, formerly known as *BotOrNot*, is a widely used social bot detection interface for Twitter [73,74,75,76].

Previous studies have figured out the different semantic features of tweet sentiments [55] and the varied social contacts [77] between human users and social bots. Based on machine learning algorithms, *Botometer* extracts more than 1000 predictive features that capture many suspicious behaviors, mainly through characterizing the account’s profile, friends, social network, temporal activity patterns, language, and sentiments [78,79]. The program then returns an ensemble classification score on a normalized scale, suggesting the likelihood that a Twitter account is a bot. Scores closer to 1 represent a higher chance of bot-ness, while those with scores closer to 0 more likely belong to humans. Congruent with the sensitivity settings of previous studies [73,74,76], we set the threshold to 0.5 to separate humans from bots. In other words, an account was labeled as a social bot if the score was above 0.5. In some cases, *Botometer* fails to output a score due to issues including account suspensions and authorization problems. Those accounts that could not be assessed were excluded from further analysis.

### 3.3. Sentiment Analysis

Although the concepts sentiment and emotion have always been used interchangeably in some studies [80], they have slight difference in their emphasis [81]. Sentiment, by default, has been defined as a positive or negative expression in NLP (Natural Language Processing) [82]. Emotion refers to diverse types of feelings or affect, such as anger, anxiety, and sadness, and emotion analysis is regarded as the secondary level of sentiment analysis in a broad sense [83]. To investigate the different sentiment and emotion components present in online messages from bots and individual accounts, we analyzed our samples with Linguistic Inquiry and Word Count (or LIWC). This automated text analysis application extracts structural and psychological observations from text records [84]. LIWC has a word dictionary composed of various linguistic categories, including a list of words that define the scale. By counting the number of words belonging to each linguistic category in each text file, LIWC returns about 90 output variables, including summary language variables, general descriptor categories, and categories tapping psychological constructs. Some of the variables include sub-dictionaries, the scale scores of which will be incremented. For instance, the word “cried” falls into five categories: sadness, verbs, negative emotions, overall affect, and past focus. Notably, LIWC2015 [85], the most recent version of the dictionary, can accommodate numbers and punctuation, as well as short phrases which are common in Twitter and Facebook posts (e.g., “:)” is coded as a positive emotions word). Previous studies have validated different indicators of the program [86,87] and our analysis mainly focused on the categories that are indicative of sentimental and emotional cues in online posts. Our study stressed the affective processes that included positive emotions and negative emotions and we specifically calculated anger, anxiety, and sadness in terms of negative emotions. Meanwhile, following previous studies [56,88,89], our study subtracted the negative score from the positive score to measure sentiments polarity and added the two scores to measure sentiments strength. Higher sentiments polarity indicates the overall sentiment that the targeted text is more positive and greater sentiments strength means more intense sentiments are expressed in the text.

### 3.4. Structural Topic Model

The topic model is a statistical model of natural language processing developed for uncovering the hidden semantic structures within large corpora [90]. It has been frequently used by computer scientists and has become increasingly popular in social science research [91]. In this study, we used the newly created Structural Topic Model (STM) to extract the latent topics in the original Twitter posts. STM is an unsupervised learning model that was introduced by Roberts et al. [92] as an extension of Latent Dirichlet Allocation (LDA) [93], one of the most common probabilistic topic models for computer-assisted text analysis. By comparing STM benefits with LDA, scholars have found that STM outperforms its predecessor model in covariate inference and out-of-sample prediction [94]. Some social scientists have used STM to analyze text from social media [95].

Before inputting the text documents, STM requires the researchers to specify a value of *k*, representing the number of topics they want to extract from the corpus. Each word that appears in the corpus has a probability of belonging to a given topic. Although the words of different topics may overlap, users can distinguish topics with the highest-ranking words. Our study used the STM R package to conduct a topic-based sentiment analysis of bot-generated and human-generated tweets. The appropriate value of *k* (the fixed number of topics) for a given corpus is user-specified, without an absolute “right” answer [96], which means that the researchers needed to set the value based on the results’ interpretability. After several preliminary tests, we initially limited the number of topics within a range of 6–14 and eventually specified k = 12. As suggested by the developers of STM [97], we labeled the topic by investigating the words associated with topics and documents associated with topics. Two functions named “labelTopics” and “findThoughts, plotQuote” in stm R package were used to generate high probability words and example documents of each topic. The STM model automatically calculated the expected topic proportions of the corpus. Moreover, for each document, the topic proportions across the *k* topics add up to 1 [97]. In this study, the topic with the largest proportion in a tweet were regarded as its dominant topic.

## 4. Results

As mentioned above, based on the timeline published by the World Health Organization [70], this exploratory study selected three data collection time points. A total of 195,201 tweets (7806, 14,375, and 173,020 at the three time points, respectively) were collected in this study, of which 18,497 (9.27%) were published by social bots, 176,166 (90.25%) by humans, and 535 (0.27%) by unknown users. A total of 118,720 unique users contributed to these tweets, of which 10,865 (9.15%) were social bots, 107,478 (90.53%) were humans, and 375 (0.32%) were unknown users.

**Basic statistics.** The STM model gave 12 lists of high probability words for 12 topics, which are displayed in descending order of proportion in Table 1. We labeled the top 11 word lists with the topic names. As the word list with the smallest proportion had no interpretable meaning, we labeled it as Else. Confirmed cases and deaths accounted for the largest proportion, followed by disease prevention and economic impacts. We further classified the top 11 topics into two categories: health-related and health-unrelated themes. Health-related themes covered eight topics, including confirmed cases and deaths, disease prevention, COVID-19 in the US, news/Q&A, COVID-19 in China, COVID-19 outbreak, healthcare system, and health risks. Health-unrelated themes included three topics, covering economic impacts, events canceled and postponed, and impacts on public life.

**Figure 1:**
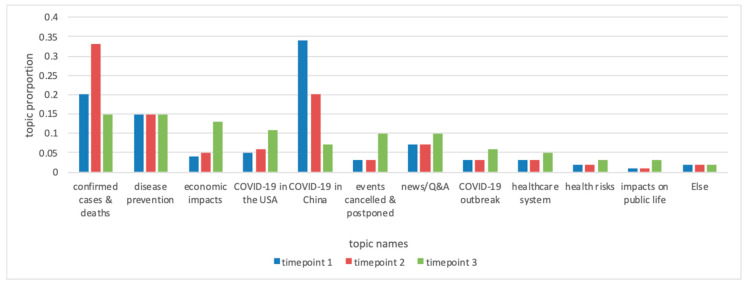
Topic proportions at the three time points.

Figure 1 shows the proportions of the 12 topics at the three time points. For each time point, the sum of the 12 topics’ proportions is 1. It should be noted that the proportions of all three health-unrelated topics climbed when the pandemic spread. As for health-related topics, Twitter users in our dataset, who are English language speakers according to our data collection criteria, showed increasing interest for COVID-19 in the US and decreasing interest for COVID-19 in China. The concern for news/Q&A, COVID-19 outbreak, healthcare system, and health risks climbed, while concern for disease prevention remained consistent. The discussions of confirmed cases and deaths reached the highest point at time point 2.

**Figure 2:**
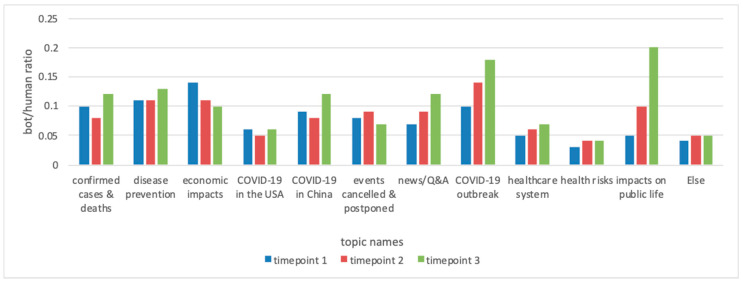
Bot/human ratio for each topic at three time points.

**Bots-human ratio in different topics at three time points.**Figure 2 shows the bot-human ratio (dividing the number of bot-generated contents by the number of human-generated contents) for every topic at each of the three time points. Tweets about economic impacts, COVID-19 outbreak, and impacts on public life received the most contributions from social bots at time point 1, time point 2, and time point 3, respectively.

Comparing the results of Figure 1 and Figure 2, we found that although the topic proportions of economic impacts increased as the pandemic developed, the bot-human ratio decreased, indicating that humans’ concern for economic impacts gradually emerged. For several health-related topics (such as news/Q&A, COVID-19 outbreak, healthcare system, and health risk) and one health-unrelated topic (impacts on public life), the increase of the topic proportion paralleled the increase of social bot involvement, suggesting social bots’ potential contributions in promoting the saliency of these topics to the social media agenda. For the topic of COVID-19 in China, social bots’ engagements increased when the topic proportions dropped at time point 3, suggesting social bots were expending effort to maintain public attention towards COVID-19 in China.

**Topic-based analysis of sentiment characteristics for social bots and humans.** In order to compare the sentiment characteristics of social bots’ contents and humans’ contents in different topics, we calculated the topic-based average value of sentiment polarity and sentiment strength for different user groups. A sentiment polarity greater than 0 refers to overall positive sentiment and polarity lower than 0 means overall negative sentiment. Greater sentiment strength means the sentiments present in texts are more intense and smaller sentiment strength means fewer sentiments are expressed. Table 2 shows the results in ascending order of overall sentiment polarity. Social bots showed similar sentiment polarity to humans in all topics. In four topics (COVID-19 in the US, health risks, COVID-19 in China, and economic impacts), more negative sentiments were expressed than positive sentiments overall and social bots were even more negative than humans when addressing COVID-19 in the US, COVID-19 in China, and economic impacts. In the other seven topics, the overall sentiment polarity was positive and social bots showed an even more positive attitude in impacts on public life, events canceled and postponed, and disease prevention. As for sentiment strength, social bots showed weaker sentiment in most topics, except for COVID-19 in the US and the healthcare system.

For the four topics of which their overall sentiment polarity was negative, we explored the expressions and human-bot interactions of three specific negative emotions in Table 3 and Table 4. Table 4 reveal the average value of sadness, anger, and anxiety for social bots and humans. When discussing COVID-19 in the US, social bots showed more anger, sadness, and anxiety than humans. When discussing COVID-19 in China, social bots showed more anger and anxiety, but less sadness. For health risks, social bots expressed more sadness and anxiety than humans, but less anger. For economic impacts, social bots showed more anger and anxiety.

Table 4 shows, for a specific kind of emotion in each topic, how much the emotions were amplified through bot-to-human retweeting, human-to-bot retweeting, bot-to-bot retweeting, and human-to-human retweeting. A total of 62,940 of the 195,201 tweets in our dataset were retweeted at least one time. These 62,940 were retweeted 416,204 times in total after they were published. We identified the retweeters of these 62,940 tweets with official Twitter API and calculated their bot score with *Botometer* to distinguish humans and suspected bots. Thus, we can classify the 416,204 instances of retweeting behavior into bot-human, human-bot, bot-bot, and human-human retweeting. Then, the scores of anger, anxiety, and sadness present in each of the 62,940 tweets were documented to calculate the volume of emotion transmission during the 416,204 retweeting behaviors. For each topic, we counted the transmission volume of sadness, anger, and anxiety through bot-bot, bot-human, human-bot, and human-human retweeting and finally calculated the emotion transmission proportions of four categories of retweeting behavior. Following the results of Table 2 and Table 3, Table 4 only shows the results of the four topics of which their overall sentiment polarity was negative.

The results shown in Table 4 reveal that emotion transmission among humans was the most intensive, while the emotion transmission among social bots was the least intensive during the pandemic. In most cases, the emotion transmission from humans to social bots was more significant than that from social bots to humans; that is, social bots were more likely to actively amplify humans’ emotions rather than to trigger humans’ amplification. Among the four topics, social bots were most capable of exporting negative emotions when talking about COVID-19 in the US. In particular, bot-to-human anger was greater than human-to-bot anger, suggesting social bots’ success in triggering anger transmission in this topic. While social bots were not capable of triggering sentiment transmission in all the topics, comparing Table 3 and Table 4, we found that although social bots expressed more sadness in response to health risks, they failed to pass sadness to humans.

## 5. Discussion

In our study, we identified the key concerns and sentiment expressions of different actors on Twitter, through which we confirmed that social bots were intentionally engaged in manipulating public opinion and sentiments during the COVID-19 crisis and that they managed to facilitate the contagion of negative emotions on certain topics.

Generally speaking, the distribution of topic proportions suggests that Twitter users reacted most actively towards the increasing infection cases and mortality, especially when the coronavirus risks spread worldwide (t2). However, unlike a previous study pointing out that the incidence of infection cases and deaths paralleled negative public sentiments [11], we found that individuals’ sentiment expression was overall positive, except when discussing the US, China, the hit to the global economy, and potential coronavirus risks. Due attention has been paid to measures for protecting people from infection, indicating that social media platforms can be effective in disease prevention. Notably, China and the US, two of the primary negative emotion triggers, turned out to be the two most prominent entities in the Twitter agenda, with a shift of focus from the former to the latter as time went by.

Regarding the role of bots in shaping online public opinion and sentiment dynamics, we pinpointed that these automated actors are influential in online social networks, either in a benign or malicious manner. Throughout all three time points that we considered, it was clear that social bots made up only a small proportion of Twitter users (9.15%) participating in COVID-19 related discussions, yet the ratio of their messages was slightly higher (9.27%). However, previous studies have indicated that social bots are often perceived as credible and attractive [98] and that by constituting 5–10% of participants involved in a discussion, these accounts can bias individual’s perceptions and dominate public opinion [99]. Our study showed that the proportion of bots at t1 and t2 remained the same, followed by a slight increase at t3. According to researchers from CMU, it was not until February that social bots started to appear in COVID-19 related conversations more frequently [65]. As shown in Table 2, congruent with previous research [55], social bots used to release weaker sentiment than human users. Our study found that the two communities shared a similar trend on sentiment polarity—positive or negative—for almost all topics, making social bots more deceptive. Through impersonating humans, these automated accounts are designed to push particular narratives and drive the discourse on social network sites [19], primarily through two approaches: content generation and interactions with human users [100].

Scholars have argued that exposure to information with “antagonistic memes”, such as those that linked Zika to global warming, can trigger polarized affections among people [101]. Social bots have been found to create fear and panic on the part of the public by means such as steering online discussions with inaccurate health claims or unverified information [102]. In our case, these malicious actors pushed discouraging and depressing messages to intensify people’s negative feelings. As these accounts facilitate the spread of misinformation by exposing human users to low-credibility content and inducing them to reshare it [76], we argue that they use emotionally-charged posts to bait humans into retweeting, thus boosting the influence of negative emotions. More importantly, since social bots have been found to retweet more than legitimate users, yet be retweeted less than them [19], our study points out that compared with the resharing-trigger strategy, social bots are even more likely to amplify pessimistic words through retweeting human users (see Table 4).

To be more specific, bots became more outraged and anxious while talking about China and the devastating blow towards the global economy, which jeopardized public impressions of these two topics. They also retweeted posts with extreme anger and angst to intensify negative emotions on Twitter. In the outbreak, human users were apt to be angry, worried, and anxious in response to health risks, and fears and actual health risks might be exaggerated while tweeting [5]. The situation was worsened due to the interference of social bots. These actors expressed even more sadness and anxiety than humans while posting, although the former failed to be transmitted from bots to human users.

Another point worth highlighting is that social bots successfully infiltrated and manipulated Twitter discourse about COVID-19 in the US. Congruent with previous research [55], our study found that social bots tended to show lower sentiments strength than human users. However, this was not the case in discussions about coronavirus and the Trump administration, when social bots posted stronger indications of anger, sadness, and anxiety compared with human users. While the negative emotions, especially anger, were inflammatory and contagious enough, human users were even more likely to amplify these messages, which might exacerbate public irritation and social conflict in the online ecosystem [21].

On a different note, in our study, social bots were also found to be increasingly active and positive in the majority of health-related discussions, including news about the outbreak, medical suggestions, and relevant information about disease prevention. On the one hand, this sheds light on the potential benefits of these automated actors. They might be leveraged by media agencies and public institutes to monitor and disseminate breaking health news, as well as to update information regarding the latest developments in disease prevention and treatment [60,61]. As COVID-19 mushroomed, people’s daily life changed markedly due to the epidemic. Our study showed that the number of social bots involved in discussions about impacts on public life increased steadily over time and the group tended to share positive messages, which might help comfort and encourage people to stay positive during the crisis. On the other hand, given the previous findings that social bots played a disproportionate role in spreading articles from low-credibility sources [76], these accounts should be treated cautiously as they may also be misleading by pretending to be positive. For instance, social bots may push narratives suggesting that the epidemic has been halted and urge public gatherings to resume. If social bots generate positive content to share information in opposition to scientific consensus, they may still pose threats to public health [102,103]. One of the negative side-effects is that the public‘s confidence in online health communication may erode [104]. Furthermore, as social media data can be used to complement and extend the surveillance of health behaviors [105], risk communicators and public health officials may fail to accurately monitor the state of emerging threats or correctly gauge public sentiments about health emergencies due to the interference of social bots [103]. Thus, the credibility and impacts of seemingly positive content need to be further examined.

## 6. Conclusions

As social media has become one of the dominant platforms for health communication in pandemics, the prevalence of social bots may potentially skew public sentiments by amplifying specific voices intentionally. This study extended previous research about social bots and sentiments by shedding light on the topic-based sentiment features of social bots in the context of health emergencies. To be specific, we characterized the differences between social bots and humans in terms of their sentiment polarity and sentiment strength and in particular, explored how negative emotions transmit among different actors.

The results indicate that suspected social bots contributed to as much as 9.27% of COVID-19 discussions on Twitter. Social bots and humans shared similar trends in sentiment polarity for almost all topics. In overall negative topics, social bots were even more negative than humans when addressing COVID-19 in the US, COVID-19 in China, and economic impacts. Social bots’ sentiment expressions were weaker than those of humans in most topics, except for COVID-19 in the US and the healthcare system. Social bots were more likely to actively amplify humans’ emotions, rather than to trigger humans’ amplification. Social bots’ capability of triggering emotion contagion varied among cases. In discussions of COVID-19 in the US, social bots managed to trigger bot-to-human anger transmission, whereas although social bots expressed more sadness towards health risks, they failed to pass sadness to humans.

### Limitations and Future Directions

This study used sentiment analysis and topic modeling to capture social bots’ sentiment engagement in distinct topics in online discussions of COVID-19, which may lay the groundwork for health researchers and communicators interested in sentiment dynamics on social media. However, although bot detection algorithms can automatically label suspected social bots, the current mechanism of the Twitter platform does not reveal the behind-the-scenes manipulators of social bots. It is challenging for researchers to demonstrate the political, social, or economic motivation of social bots. Thus, in this study, although we depicted the sentiment features of social bots and put forward several probable explanations, we cannot conclude the intention of social bots based on their sentiment expressions. In this study, we also explored bot–human sentiment transmission in one-step retweet cascades. Social media is a complex information eco-system, so social bots and humans are actors embedded in large-scale social networks. In future studies, a network perspective could be introduced to identify social bots’ roles in starting, promoting, hindering, or terminating sentiment transmission. Sentiment transmission networks of different sizes and patterns could be characterized to extend the current understanding of social bots’ impacts on online sentiments.

## Figures and Tables

**Table 1:** List of 12 tweet topics according to the structural topic model.

Name	Category	Proportion	High Probability Words	Example Tweet
Confirmed cases & deaths	Health-related	0.16	coronavirus, case, health, Italy, death, confirmed, state, emergency, number, update, breaking, country, positive, public, total	Colorado reports 16 new cases of #CoronaVirus: Breakdown: Arapahoe County: 3 + 1 Jefferson County: 3 + 2 Pitkin County: +9 Larimer County: 1Gunnison County: 2 + 1 Denver County: 6 + 4 (plus 1 indeterminate) Douglas County: 3 Eagle County: 4 + 1 El Paso County: 1 Summit County: 1
Disease prevention	Health-related	0.15	coronavirus, virus, corona, hand, don’t, stay, keep, wash, safe, mask, face, hope, protect, avoid, kill	# coronavirus #prevention # coronavirus #prevention #HealthyLiving “Cover your mouth and nose with a tissue when you cough or sneeze, then throw the tissue in the bin and wash your hands. If you do not have a tissue to hand, cough or sneeze into your elbow rather than your hands.
Economic impacts	Health-unrelated	0.12	coronavirus, work, business, sick, crisis, plan, government, online, market, student, budget, economy, class, employee, impact	The Chancellor @RishiSunak sets out a £30 bn #coronavirus plan: Sick pay for self-employed + help on UC £500 m hardship fund No cost of sick pay to SMEs £1 bn of working capital loans. No Rates for small hospitality biz £3000 grant to small businesses #Budget2020
COVID-19 in the US	Health-related	0.11	coronavirus, trump, cant, real Donald Trump, american, toilet, going, paper, president, house, america, word, medium, ready, thread	When you have nothing else. IDENTITY POLITICS! You & your party are divisive & insane. #CoronaVirus #BLEXIT #WalkAway #MAGA #WWG1WGA #TRUMP #TheGreatAwakening #TRUTH #DNC #Democrat #MSM #IdentityPolitics #FearMonger
COVID-19 in China	Health-related	0.09	china, coronavirus, spread, Wuhan, virus, travel, outbreak, country, quarantine, Chinese, measure, infected, hospital, flight, city	After the spread of a new #Coronavirus, the #UK is taking precautionary measures by monitoring all flights arriving from China. The measures will be applicable on flights from Wuhan to London Heathrow, where aircraft will land in an isolated part of Terminal 4.
Events canceled & postponed	Health-unrelated	0.09	coronavirus, event, canceled, year, game, going, cancel, march, concern, fan, big, canceled, postponed, decision, closed	Big West Basketball No spectators will attend tournament due to #coronavirus concerns. Honda Center Games (no fans) Thu—Men’s quarterfinal games Fri—Men’s and women’s semifinals Sat—Both championship games #AnaheimSports @HondaCenter
News/Q&A	Health-related	0.09	coronavirus, news, read, help, great, question, community, latest, expert, watch, situation, free, advice, outbreak, video	Tonight at 8 p.m. ET on Tonight at 8 p.m. ET on @NBCNewsNOW: @DrJohnTorres hosts special coverage to answer questions about #coronavirus. Stream it live tonight on @Roku, @amazonfiretv, @AppleTV and http://nbcnews.com/NOW
COVID-19 outbreak	Health-related	0.06	coronavirus, Covid, coronavirus outbreak, well, real, feel, worse, Corona virus UK, officially, Coronavid, corona virus USA, deal, coviduk, corona outbreak, epidemic	From the air, hunger, fire and war, save us, Lord “Pray for Italy & the World! Today at 8 p.m. #everyday, the supplications will also be sung, begging for the protection against the #coronavirus epidemic
Healthcare system	Health-related	0.05	pandemic, coronavirus, Corona virus update, testing, test, disease, CDC, healthcare, system, vaccine, patient, classifies, spread, action, outbreak	#Coronavirus Pandemic: Declared as #pandemic by World Health Organization WHO deeply concerned by alarming levels of spread & severity, and alarming levels of inaction In US, slow rollout of testing (flawed kits) and limited testing capacity crippled response to #COVID19
Health risks	Health-related	0.03	coronavirus, care, risk, panic, better, lot, family, life, serious, bad, person, friend, told, symptom, ill	The virus can remain intact at 4 degrees (39 degrees Fahrenheit) or 10 degrees (50 F) for a longer period of time” Nicholls said, referring to Celsius measurements, according to the transcript. “But at 30 degrees (86 degrees F) then you get inactivation #Coronavirus
Impacts on public life	Health-unrelated	0.03	coronavirus, day, school, week, close, spreading, fast, social, shut, hour, conference, control, ago, open, closing	Close the schools in areas effected by the #coronavirus. Close the schools. Close the schools. Close the schools. Close the schools. Close the schools. Close the schools. Close the schools. Close the schools. Close the schools. Close the schools. Close the schools. Close them now
Else	Else	0.02	people, time, good, flu, thing, today, month, start, call, coming, thought, best, long, seriously, story	#coronavirus freestyle @officialnairam1 @olamide_YBNL @DONJAZZY @davido @Tecknoofficial @iamkissdaniel @lilkeshofficial @mayoku

**Table 2:** Sentiments polarity and strength of all users, social bots, and humans for different topics.

	Sentiments Polarity			Sentiments Strength		
	All Users	Social Bot	Humans	All Users	Social Bot	Humans
COVID-19 in the US	−2.15	−2.22	−2.15	4.88	4.95	4.88
Health risks	−1.84	−1.79	−1.84	5.44	5.36	5.44
COVID-19 in China	−0.72	−0.95	−0.69	2.77	2.7	2.78
Economic impacts	−0.07	−0.28	−0.04	3.4	3.31	3.41
Healthcare system	0.28	0	0.3	2.61	2.66	2.61
Else	0.42	0.87	0.4	4.73	4.58	4.74
Confirmed cases and deaths	0.46	0.26	0.49	1.63	1.13	1.69
Impacts on public life	0.51	1.87	0.24	2.36	2.05	2.43
Events canceled & postponed	0.76	0.95	0.75	3.26	2.86	3.29
Disease prevention	0.97	1.47	0.9	4.9	4.78	4.92
COVID-19 outbreak	1.21	0.65	1.31	2.93	2.44	3.02
News/Q&A	3.28	2.69	3.34	3.63	3.41	3.66

**Table 3:** Sadness, anger, and anxiety of social bots and humans for topics of which their overall sentiments polarity was negative.

Topics	Sadness		Anger		Anxiety	
	Bot	Human	Bot	Human	Bot	Human
COVID-19 in the US	0.82	0.78	2.3	2.12	1.42	0.78
Health risks	1.08	0.78	0.5	0.74	2.83	0.78
COVID-19 in China	0.4	0.46	1.14	1.02	0.79	0.46
Economic impacts	0.49	0.58	0.62	0.59	1.14	0.58

**Table 4:** Emotion transmission among different user groups for four topics of which their overall sentiments polarity was negative.

Topics	Emotions	Bot-To-Human	Human-To-Bot	Bot-To-Bot	Human-To-Human	Total
COVID-19 in the US	sadness	0.12	0.12	0.03	0.73	1
	anger	0.12	0.1	0.03	0.74	1
	anxiety	0.07	0.12	0.02	0.79	1
Health risks	sadness	0	0.06	0	0.94	1
	anger	0	0.05	0	0.95	1
	anxiety	0.05	0.06	0.01	0.88	1
COVID-19 in China	sadness	0.06	0.08	0.02	0.84	1
	anger	0.03	0.12	0.01	0.84	1
	anxiety	0.01	0.12	0	0.87	1
Economic impacts	sadness	0.02	0.09	0.01	0.89	1
	anger	0.06	0.1	0.01	0.83	1
	anxiety	0.04	0.11	0.01	0.84	1
